# Requirement of a complex motor task to identify neuroplastic changes in motor control of the lower extremity in patients with anterior cruciate ligament reconstruction: a fNIRS study

**DOI:** 10.3389/fnhum.2025.1595284

**Published:** 2025-07-10

**Authors:** Ke Liu, Qin Zhu, Weidong Xu

**Affiliations:** ^1^College of Exercise and Health, Shanghai University of Sport, Shanghai, China; ^2^Division of Kinesiology and Health, University of Wyoming, Laramie, WY, United States; ^3^Department of Orthopedics, Changhai Hospital, The Navy Medical University, Shanghai, China

**Keywords:** anterior cruciate ligament reconstruction, real motor task, neuroplastic, difficulty adaptation, functional near-infrared spectroscopy

## Abstract

**Introduction:**

Neuromuscular control is a crucial component in restoring dynamic joint stability following anterior cruciate ligament reconstruction (ACLR). The central nervous system, as the primary control center, is known to exhibit neuroplastic changes. However, motor tasks used to assess brain function in ACLR are often limited to simple and static movements. The current study aimed to compare brain activation between patients with ACLR (ACLR group) and healthy controls (CONT group) during both simple and complex motor tasks and to examine the relationship between brain activity and clinical functions to explore the underlying mechanisms of neuroplasticity.

**Methods:**

A total of 35 patients with ACLR and 25 healthy controls participated in this study. Functional near-infrared spectroscopy was used to capture real-time brain activation during knee flexion-extension (K-FE) and single-leg squat (SLS) tasks. Clinical assessments included quadriceps strength, single-leg hop, and self-reported functional outcomes. A two-way mixed-design ANOVA was conducted with one between-subject factor (group) and one within-subject factor (task). The dependent variable was the change in oxyhemoglobin concentration (ΔHbO) across six brain regions.

**Results:**

For the affected limb tasks, the Primary Somatosensory Cortex (S1) and Supramarginal Gyrus (SMG) showed significant main group effects (*P*_S1_ = 0.035, *P*_SMG_ = 0.002), whereas all brain regions showed significant main effects of task difficulty. A significant interaction between group and task was observed in the SMG (*p* = 0.036). For the contralateral limb tasks, no significant main effect of group or task was found across all brain regions. Pre-Motor Cortex (PMC), S1, Frontal Eye Fields (FEF), and SMG showed significant interaction effects between group and task (*P*_PMC_ = 0.013, *P*_S1_ = 0.015, *P*_FEF_ = 0.015, and *P*_SMG_ = 0.018). Multiple negative correlations were found between increased ΔHbO and functional outcomes in various brain regions, depending on the limb and task.

**Conclusion:**

Brain activation increased with task difficulty. Patients with ACLR showed lower somatosensory cortex activation during affected limb tasks. Their task adaptation was weaker than that of healthy controls, suggesting deficits in proprioception and a lack of neural resources for adaptation to task complexity. The significant interaction effects observed during the contralateral limb tasks indicated the compensatory role of the contralateral limb. These conclusions were supported by correlations with clinical outcomes.

## 1 Introduction

Anterior Cruciate Ligament (ACL) injuries are the most common ligament injuries of the lower extremity, with a worldwide pooled incidence of 17.5 injuries per 100,000 person-years (Ponkilainen et al., 2022). The ACL plays a critical role in maintaining anterior knee stability. As a result, injury to the ligament compromises joint mechanics, muscular strength, and overall joint function and can result in knee pain and effusion (Filbay and Grindem, 2019). ACL reconstruction (ACLR) is a standard surgical procedure that reconstructs the ligament to restore the stability of the knee joint. With over 30 years of surgical development, reconstruction surgery has become an effective treatment to relieve pain and restore static knee stability (Diermeier et al., 2021), and it is chosen by approximately 75% of patients (Cevallos et al., 2021).

Despite ACLR, patients still face a higher risk of re-injury (Wiggins et al., 2016) and early-onset post-traumatic osteoarthritis (PTOA; Liukkonen et al., 2023) than healthy individuals, and return-to-sport rates remain suboptimal (Lai et al., 2018; Brzeszczyński et al., 2022). These functional impairments may be due to impaired dynamic joint stability, resulting from persistent neuromuscular control deficits post-injury or surgery (Wikstrom et al., 2006). Such deficits may be induced by mechanoreceptor damage, pain, and inflammation after ACL injuries, tissue trauma, peripheral nerve block during surgery, and compensatory motor strategies in the post-rehabilitation program, which will affect normal dynamic joint stability and heighten injury risk during sport (Criss et al., 2021). Previous evidence has identified neuromuscular control deficits in peripheral afferent input and motor output, including but not limited to decreased somatosensory evoked potentials (SEP; Valeriani et al., 1996) and voluntary activation (Tayfur et al., 2021). As the governor of the sensorimotor system, the central nervous system also undergoes changes due to altered afferent input and feedback mechanisms (Neto et al., 2019; Tayfur et al., 2021). Increasing evidence revealed that motor, sensory, and cognitive cortices in patients with ACLR showed significant differences compared to healthy controls during motor tasks (Grooms et al., 2017; Lepley et al., 2019; Criss et al., 2020). However, depending on the selected motor task, the activation location or level of functional brain areas varies (An et al., 2019; Jiganti et al., 2020; Kim et al., 2023; Sherman et al., 2023). Functional magnetic resonance imaging (fMRI) and electroencephalography (EEG) are commonly used to observe changes in brain activation. However, their inherent limitations restrict their use in dynamic motor tasks. fMRI is limited by poor temporal resolution, whereas EEG offers high temporal resolution but low spatial resolution. In addition, both techniques are vulnerable to motion artifacts, particularly during complex or full-body movements, which limits their use in simple motor tasks such as knee flexion/extension (Cutini and Brigadoi, 2014). Brain activation during complex and realistic motor tasks still needs to be explored (Miao et al., 2017).

Functional near-infrared spectroscopy (fNIRS) is a neuroimaging technique using optical sensors placed on the scalp surface to capture the brain hemodynamics, which offers a portable solution for quantifying the brain activation (Kohl et al., 2020). fNIRS offers temporal and spatial resolutions that fall in between fMRI and EEG, balancing the trade-off between temporal and spatial resolutions in quantifying the changes in brain activation (Lloyd-Fox et al., 2010). Such an advantage, along with its portability, makes fNIRS a promising technique for studying brain activity during dynamic motor tasks. Previous studies have proved that the fNIRS outcome has a strong correspondence with the fMRI during a motor task (Zinos et al., 2024). Hence, this study employed fNIRS to explore the brain activation differences between patients with ACLR and healthy individuals during both simple-static and complex-dynamic tasks. In the meantime, clinical functions were assessed to correlate with cortical activity to explore the mechanisms underlying neuroplastic changes. We hypothesized that there would be differences in brain activation as a function of group and task. Each group was expected to exhibit distinct brain activation patterns in response to task difficulty, which can be explained by their correlation with clinical functional outcomes.

## 2 Participants and methods

### 2.1 Experimental design

This study used a cross-sectional exploratory design to compare variables between the two groups. The independent variables included one between-group factor [group: ACLR group, healthy controls (CONT group)] and one within-subject factor [task difficulty: knee flexion-extension task (K-FE), single leg squat (SLS)]. The dependent variables were changes in hemoglobin in the detected brain areas and clinical functional outcomes.

### 2.2 Participants

The ACLR group was recruited from patients who underwent ACLR at Changhai Hospital via an invitation flyer. To be eligible for the study, the patient must be 18–45 years old, have sustained a unilateral ACL injury, undergone reconstruction surgery, and have completed at least 3 months of recovery after surgery. Patients with a history of other lower-limb surgeries or multi-ligament knee injuries were excluded. Participants with severe pain or fluid in their knees before the formal test were also excluded. The CONT group included participants without a history of severe musculoskeletal injuries or lower-limb surgery. The dominant leg was defined as the limb used to kick the ball.

The exclusion criteria for all participants were as follows: (1) confirmed neurological or psychiatric illness; (2) dominant hand is the left hand; (3) acute musculoskeletal injury to other lower-limb joints within the past 3 months, resulting in at least one missed day of physical activity; (4) diagnosed balance or vestibular disorder affecting the body balance; (5) use of medications affecting cognitive functions; (6) pregnancy.

From December 2023 to September 2024, 44 patients with ACLR (25 left and 19 right) and 27 healthy participants were enrolled. Although participants were recruited with the aim of matching group sizes, the final sample differed due to eligibility screening and poor-quality brain signal recordings. Three patients with ACLR (three left) were excluded due to not meeting eligibility criteria, six patients with ACLR (four left, two right), and two healthy subjects were excluded due to inadequate brain signal quality. *A priori* power analysis using G*power software indicated that a minimum of 46 participants were required to detect a moderate effect at *p* = 0.05 with 90% power. Ultimately, 35 patients with ACLR (18 left, 17 right) and 25 healthy controls were included in the study.

The study was registered in the Chinese Clinical Trial Register portal (ChiCTR2400086199) and was approved by the Ethics Committee of Shanghai University of Sport (102772024RT063). All methods were conducted in accordance with the latest guidelines and regulations of the Declaration of Helsinki.

### 2.3 Experimental procedure

Participants were asked to get ample sleep, avoid alcohol, caffeine, or other stimulants within 24 h of testing, and arrive with clean, dry, product-free hair. Before testing, the study’s purpose and procedures were explained, and written informed consent was obtained. To prevent the influence of clinical function tests on brain activation during motor tasks, the testing was conducted in a standardized order as follows: cortical activation during motor tasks, single-leg hop, muscle strength, and patient-reported outcomes. All tests were completed in a single session.

### 2.4 Data acquisition

#### 2.4.1 fNIRS data acquisition and motor tasks

**Figure 1:**
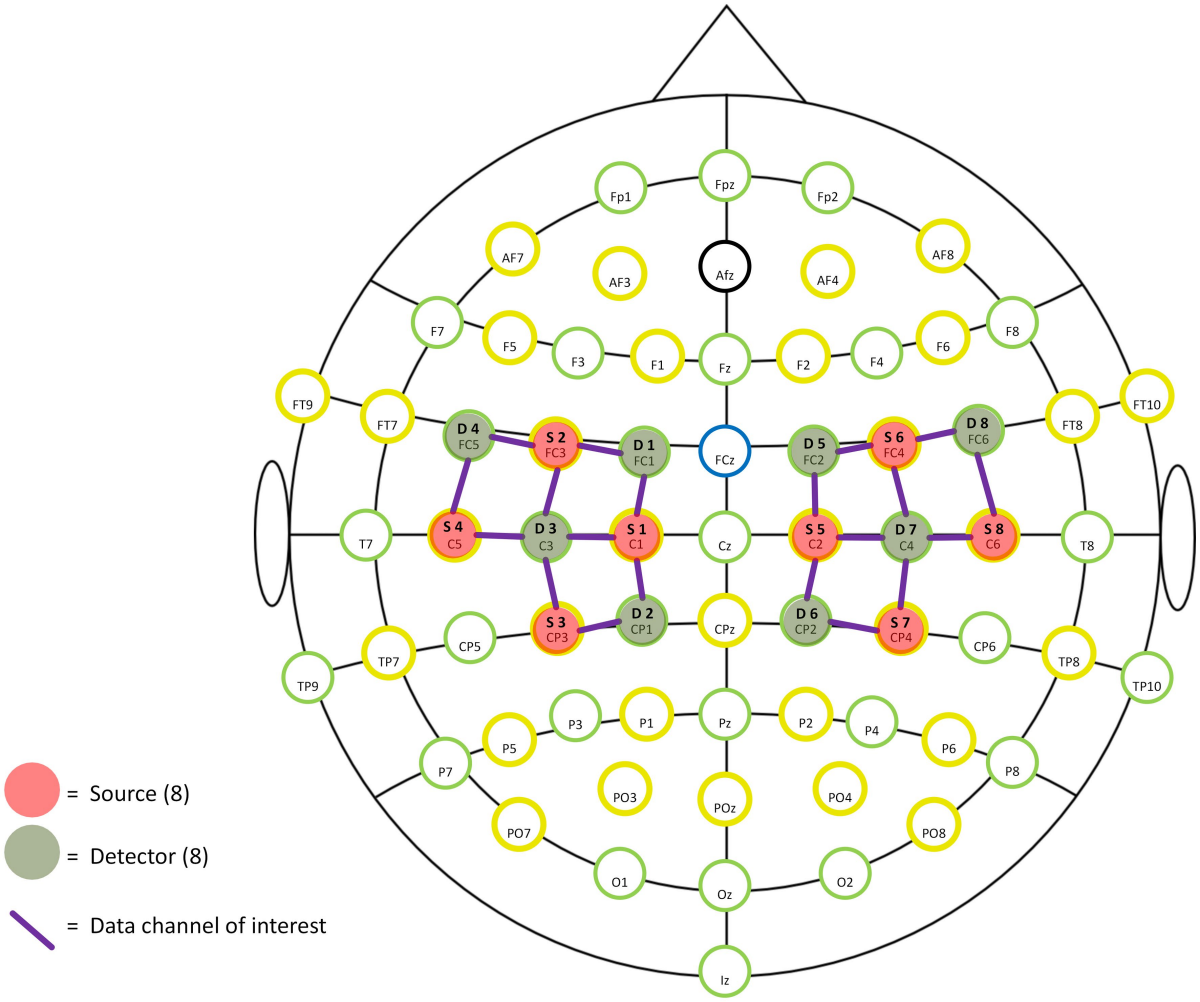
Specific optodes and channel locations (2D).

A portable near-infrared brain imaging system (NIRSport 2, NIRx, United States) was used to record hemodynamic signals from localized brain regions during motor tasks. The device included 8 emitters and 8 detectors, arranged to cover both hemispheres with 20 connected channels (see Figure 1). The inter-optode distance was 3 cm, and the wavelengths were 760 and 850 nm, with a sampling frequency of 10.2 Hz.

**Figure 2:**
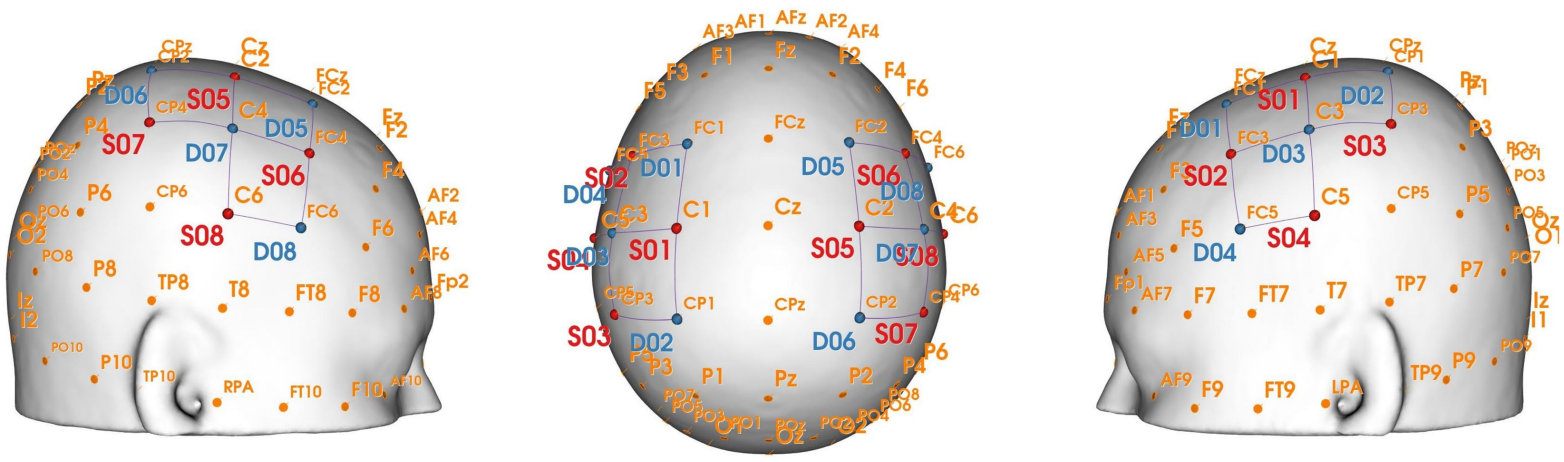
Specific optodes and channel locations (3D).

Based on the fNIRS Optode Location Decider (fOLD) toolbox and the number of optodes, the configuration was positioned to overlay the sensorimotor cortical areas (Jurcak et al., 2007; Zimeo Morais et al., 2018). Specific locations of optodes and channels are shown in Table 1 and Figures 1, 2. This configuration included the Pre-Motor and Supplementary Motor Cortex (PMC): ch1, ch3, ch5, ch10, ch11, ch13, ch15, ch20; Primary Somatosensory Cortex (S1): ch2, ch9, ch12, ch19; Includes Frontal eye fields (FEF): ch4, ch14; Prefrontal cortex (PFC): ch6, ch16; Somatosensory Association Cortex (SAC): ch7, ch17; Supramarginal gyrus (SMG): ch8, ch18. Supplementary Table 1 shows the Montreal Neurological Institute (MNI) coordinates and corresponding Brodmann area for each optode and channel, which were calculated using NIRS-SPM.

**Table 1:** Specific optode locations.

Sources	Location in 10–10 system	Detectors	Location in 10–10 system
S01	C1	D01	FC1
S02	FC3	D02	CP1
S03	CP3	D03	C3
S04	C5	D04	FC5
S05	C2	D05	FC2
S06	FC4	D06	CP2
S07	CP4	D07	C4
S08	C6	D08	FC6

An elastic head cap with the international 10–10 system for EEG was used to ensure consistency in the placement of the optode by adjusting the size and shape for each subject’s head. The cranial vertex (Cz) was used as a marker to place the cup, which was located at the intersection of the line between the preauricular points and the line from the nasion to the inion. Additionally, the alignment along the midsagittal plane was visually checked. The fNIRS optodes were held by the cap, fixed by two bands surrounding the subject’s head. Before data collection from each participant, the hair beneath the optodes was parted, and the optodes were covered with an opaque black cloth to ensure the signal quality of each channel.

**Figure 3:**
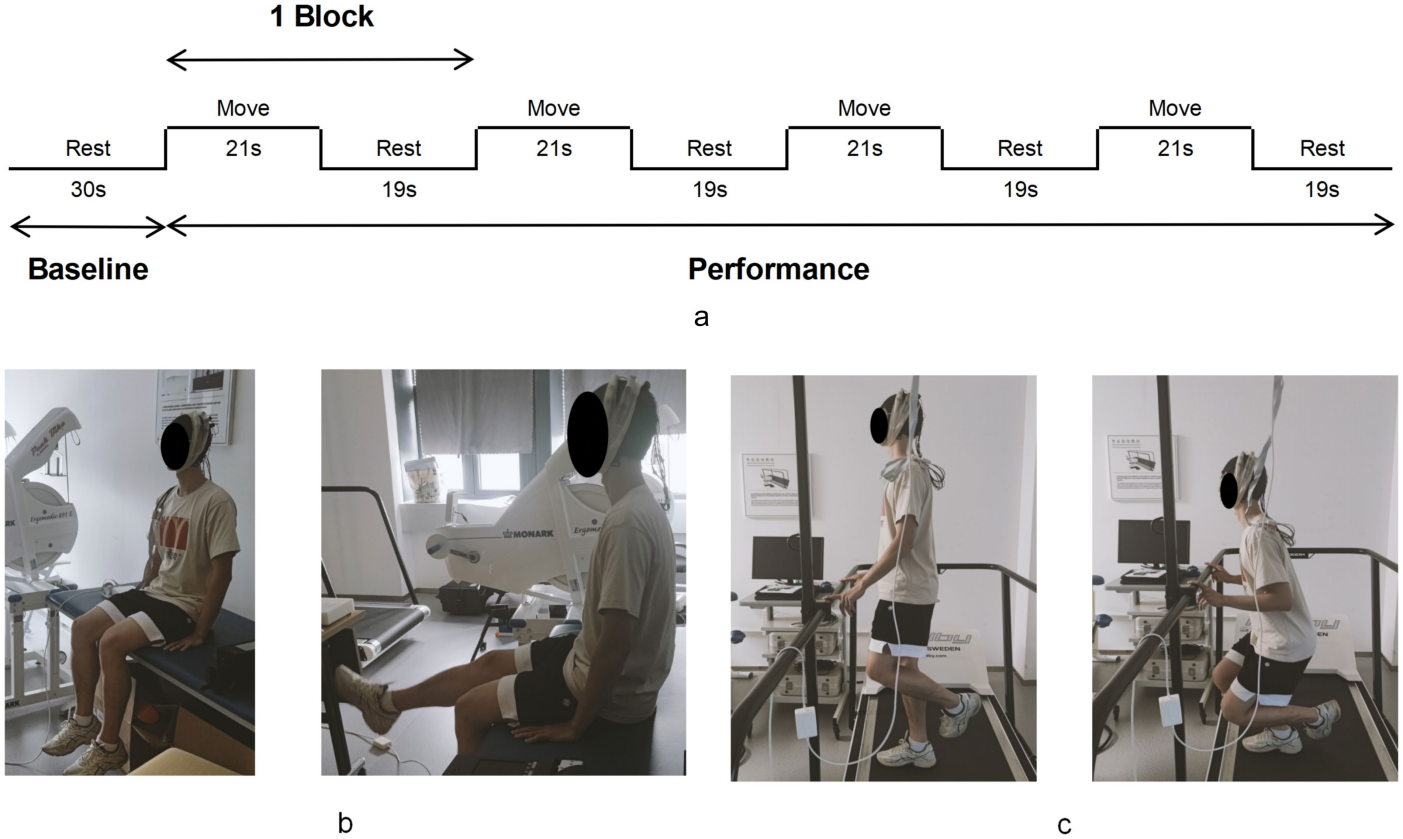
The motor tasks process. The block paradigm of motor tasks **(a)**. The knee flexion-extension task **(b)** and the single-leg squat **(c)**.

Participants sequentially accomplished two motor tasks while fNIRS signals were measured: knee flexion-extension task (K-FE) and single-leg squat (SLS). Both motor tasks were executed in the same block paradigm, consisting of the baseline state (30s) and performance state (160 s), as shown in Figure 3a (Yücel et al., 2021). During K-FE (Figure 3b), the participants were asked to sit on the bed, with hands placed at the body’s side, hip and knee flexed at 90°, naturally in the baseline state; while in the performance state, they completed 4 trials of 21 s knee flexion/extension with 19 s intervals in between trials for rest, therefore, a total of 160 s long block. Audio instructions were provided during the trial. Upon hearing the “ding” signal, participants began extending their legs to 0°, followed by flexing back to 90° within 3 s. During the interval rest, participants performed the same as in the baseline state. During SLS (Figure 3c), the baseline state required the participants to stand on the platform with both feet as normal and rest two fingertips on the front handrail to help maintain balance (Ageberg and Cronström, 2018). Then, the same procedure described in the performance state of K-FE was repeated, except that the participant lifted and bent the non-testing leg at 90° before 2 s of every block started, then squatted the testing leg as deep as possible by keeping the trunk upright and standing up within 3 s upon hearing a “ding” signal. Participants performed the same as the baseline state during interval rest. The fNIRS signal records of each task were initiated with an E-Prime program simultaneously, which played the audio instruction and marked the start and end of each block in the fNIRS records in sync. For each task, the contralateral leg was assessed first, followed by the affected leg. To prevent fatigue, a 3–5 min rest period was provided between the two tasks.

All procedures were performed in a dim and quiet environment to prevent external interruptions that could affect participants’ focus and performance. Additionally, to minimize the impact of motion artifacts, participants were asked to maintain a stable head posture and limit any unnecessary movement, such as clenching teeth or facial expressions, during the testing process. Prior to the formal test, two or three practice block trials were allowed. If participants made mistakes or lost balance during motor tasks, data were discarded, and the trial was repeated. Researchers monitored and assisted participants to ensure safety and protocol adherence.

#### 2.4.2 Single-leg hop

The single-leg hop test was used to evaluate dynamic knee stability (Fitzgerald et al., 2001). Participants stood behind a starting line and hopped forward on the testing leg. Trials were valid if the participant maintained balance for 2 s without touching down with the other foot or hands (Gustavsson et al., 2006). Practice attempts were allowed before the formal test. Three valid trials were performed for each leg, starting with the contralateral leg.

#### 2.4.3 Isokinetic strength acquisition

Quadriceps strength was measured using an isokinetic dynamometer (Con-Trex® MJ; Physiomed, Germany) following a validated protocol (Maffiuletti et al., 2007). Participants were secured into an isokinetic dynamometer using shoulder and lap straps, with their hips and testing knees secured at 90° of flexion. During the test, participants were instructed to hold on to the handles beside the chair. The alignment between the dynamometer rotational axis and the knee joint rotation axis (lateral femoral epicondyle) was checked at the beginning of each trial. The gravity effect torque was corrected in each participant throughout the range of motion. In the formal test, participants performed five trials of isokinetic maximal voluntary contractions in the range of 15°-80° of knee flexion at a speed of 60°/s for each leg. As part of a standardized procedure, participants were allowed to practice one trial prior to the formal test. The researchers delivered consistent verbal encouragement during the formal test to encourage maximal muscle strength production. The contralateral leg was tested before the affected leg.

#### 2.4.4 Subjective knee evaluation

Participants completed two questionnaires, including the International Knee Documentation Committee (IKDC) form and ACL Return to Sport Index (ACL-RSI) form. The IKDC assesses knee-specific function and disability, with higher scores indicating better recovery (Higgins et al., 2007). The ACL-RSI evaluates psychological readiness, which includes emotions, confidence, and perceived risk. It ranges from 0 to 120, with higher scores reflecting greater readiness to return to sport (Jia et al., 2018).

### 2.5 Data processing

#### 2.5.1 Hemodynamics

Data preprocessing was conducted using the Homer2 toolbox in Matlab (R2013b, MathWorks Inc., Natick, United States) following a standardized order (Gemignani and Gervain, 2021). Raw data were first calculated for the coefficients of variation (CV, %) of the dual-wavelength raw intensity signals to check the signal quality of each channel. Based on these calculations, channels with a CV > 15% or trials with a CV > 10% were rejected and removed. The optical intensity data were then converted into optical density data. Artifact correction was detected (tMotion = 0.5 s, tMask = 1 s, SDThresh = 8, AMPThresh = 5) and corrected by linear interpolation and Wavelet (Cooper et al., 2012). Subsequently, the signal was filtered by a 0.01 Hz and 0.03 Hz bandpass, which is approximately the frequency of hemodynamic changes in the experimental block (Pinti et al., 2018). Using the modified Beer–Lambert Law, optical density data were converted to the concentrations of oxyhemoglobin (HbO) and deoxyhemoglobin (HbR). While the former is sensitive to the 830 nm near-infrared light, the latter is sensitive to the 780 nm near-infrared light. Because of the higher signal-to-noise ratio and higher reproducibility of HbO, HbO data were chosen (Kinder et al., 2022). Since the baseline concentration of HbO varied for different people, the mean value of HbO within the 5 s before each stimulus onset was selected as the baseline for HbO correction of each block, resulting in the magnitude of HbO changes (ΔHbO, μmol/L). The 5–20s ΔHbO after every onset of the block was averaged for each channel, which represented the magnitude of neural activation. The value of task-evoked functional brain activity in each brain area was used for the final statistical analysis, which was quantified by averaging the block-averaged ΔHbO of the included channels.

#### 2.5.2 Isokinetic strength limb symmetry index

The mean isokinetic peak torque (Nm) was calculated by averaging the peak torque of five trials. The quadriceps limb symmetry index (Q-LSI) was calculated as the ratio of the mean isokinetic peak torque of the quadriceps between limbs (affected limb/contralateral limb) × 100%, which was used for the final correlation analysis.

#### 2.5.3 Single-leg hop distance limb symmetry index

The test outcome was measured as the distance from the starting line to the heel in the landing position, accurate to 0.1 m. Then, the single-leg hop distance limb symmetry index (SLHD-LSI) was calculated as the ratio of the mean distance of three trials between limbs (affected limb/contralateral limb) × 100%, which was used for the final correlation analysis.

### 2.6 Statistical analysis

Statistical analysis was performed using IBM SPSS Statistics 20.0. The normality of the demographic, brain activity, and functional outcomes was confirmed using the Shapiro–Wilk test. Independent sample t-tests and Mann–Whitney U tests were used to compare baseline demographic variables between groups. Specifically, independent t-tests were used for normally distributed variables (e.g., weight, post-surgery Tegner score), and Mann–Whitney U tests for non-normally distributed variables (e.g., age, weight, pre-injury Tegner score). Categorical variables, such as gender and dominant leg distribution, were compared using chi-squared tests.

For brain activation outcomes (ΔHbO), two-way mixed-design ANOVAs were performed with one between-subjects factor (groups: ACLR, CONT) and one within-subjects factor (task: K-FE, SLS), separately for each limb. When interaction effects were significant, simple effects analyses were conducted with Bonferroni correction. If normality assumptions were violated, Generalized Linear Models (GLMs) were used to assess main and interaction effects instead.

Pearson’s or Spearman’s correlation analyses (depending on normality) were used to examine associations between cortical activity and functional outcome measures. For all analyses, the level of significance was set at *p* < 0.05.

## 3 Results

### 3.1 Demographic data

Table 2 summarizes the demographic data of the participants in both groups. In the ACLR group, three types of grafts were included, and the mean time since surgery was 7.44 ± 3.59 months. There were no significant differences between groups in age, gender, height, weight, dominant leg, or pre-injury Tegner (*p* > 0.05). However, the post-surgery Tegner score was significantly lower in the ACLR group than in the CONT group (*t* = 3.526, *p* = 0.001), indicating reduced physical activity among patients with ACLR.

**Table 2:** The demographic of the participants (mean ± SD).

Characteristics	ACLR group (*n* = 35)	CONT group (*n* = 25)	t/*Χ*^2^	*p*
Injury side (left/right)	18/17			
Graft type: LARS/PLT/HT	16LARS /7GT /12HT			
Time post-surgery (month)	7.44 ± 3.59			
Age (year)	29.6 ± 8.37	25.87 ± 3.20	−1.744	0.081
Gender (male/female)	24/11	17/8	0.002	0.963
Height (cm)	174.4 ± 8.67	173.09 ± 8.21	−0.876	0.381
Weight (kg)	75.02 ± 14.22	69.43 ± 12.72	−1.525	0.133
Dominant Leg (left/right)	2/33	1/24	0.09	0.764
Tegner activity scale (pre-injury score)	6.66 ± 2.33	6.04 ± 1.55	−1.199	0.231
Tegner activity scale (post-surgery score)	4.34 ± 1.94	6.04 ± 1.55	3.526	0.001*

ACLR, anterior cruciate ligament reconstructed patients; CONT, the healthy control subjects; HT, hamstring tendon graft; PLT, peroneus longus tendon graft; LARS, artificial ligament graft; *means significant difference.

### 3.2 ΔHbO in different brain areas as a function of group and task

**Figure 4:**
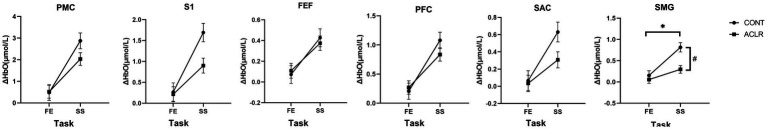
The group × task interaction effects in ΔHbO of different brain areas during affected limb tasks. PMC, Pre-Motor and Supplementary Motor Cortex; S1, Primary Somatosensory Cortex; FEF, Frontal Eye Fields; PFC, Prefrontal Cortex; SAC, Somatosensory Association Cortex; SMG, Supramarginal Gyrus. *indicates a significant group and task interaction effect. # indicates significant group differences within a task.

Table 3 presents the main effects of group and task, as well as their interaction on ΔHbO across different brain regions during the task with the affected limb. Significant group main effects were observed in the S1 and SMG regions, where the CONT group showed greater ΔHbO compared with the ACLR group (S1: [0.98 ± 0.16 vs. 0.56 ± 0.13] μmol/L; SMG: [0.48 ± 0.08 vs. 0.18 ± 0.06] μmol/L). No other brain areas exhibited significant group effects. A significant main effect of task was found across all six regions, with SLS producing greater ΔHbO values than K-FE (PMC: [2.45 ± 0.23 vs. 0.50 ± 0.24] μmol/L; S1: [1.30 ± 0.14 vs. 0.24 ± 0.14] μmol/L; FEF: [0.40 ± 0.06 vs. 0.09 ± 0.06] μmol/L; PFC: [0.96 ± 0.09 vs. 0.24 ± 0.09] μmol/L; SAC: [0.47 ± 0.07 vs. 0.05 ± 0.07] μmol/L; SMG: [0.56 ± 0.07 vs. 0.10 ± 0.07] μmol/L). A significant group × task interaction effect was observed only in SMG (Figure 4). Simple effects analysis revealed that the ΔHbO difference between groups was significant in the SLS task (*p* < 0.001), with greater activation in the CONT group (0.81 ± 0.11 vs. 0.30 ± 0.09 μmol/L). No significant group differences were observed during the K-FE task (*p* = 0.503).

**Table 3:** Results of two-way mixed ANOVA for ΔHbO across brain regions during affected limb tasks.

	Main group effect		Main task effect		Group × Task interaction effect	
	*F*	*p*	*F*	*p*	*F*	*p*
PMC	1.472	0.225	34.534	<0.001*	1.752	0.186
S1	4.432	0.035*	27.776	<0.001*	3.398	0.065
FEF	0.011	0.916	15.501	<0.001*	0.314	0.576
PFC	0.504	0.478	31.988	<0.001*	1.457	0.227
SAC	2.862	0.091	15.917	<0.001*	1.917	0.166
SMG	9.267	0.002*	20.082	<0.001*	4.389	0.036*

*indicates significant difference.

**Figure 5:**
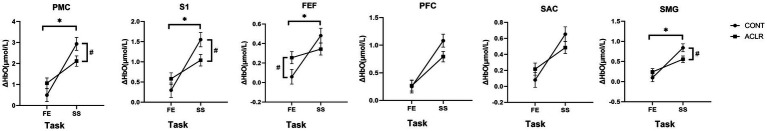
The group × task interaction effects in ΔHbO of different brain areas during contralateral limb tasks. PMC, Pre-Motor and Supplementary Motor Cortex; S1, Primary Somatosensory Cortex; FEF, Frontal Eye Fields; PFC, Prefrontal Cortex; SAC, Somatosensory Association Cortex; SMG, Supramarginal Gyrus. *indicates significant group and task interaction effect. # indicates significant group differences within a task.

Table 4 shows the same analysis performed during the task using the contralateral limb. There were no significant main effects according to group, indicating similar ΔHbO between ACLR and CONT groups. Specifically, all *p*-values for group effects were greater than 0.44 across regions, indicating no meaningful between-group differences during the contralateral limb tasks. However, task effects were significant across all brain regions again, showing greater activation during SLS compared to K-FE (PMC: [2.53 ± 0.197 vs. 0.78 ± 0.198] μmol/L; S1: [1.30 ± 0.12 vs. 0.44 ± 0.12] μmol/L; FEF: [0.41 ± 0.05 vs. 0.16 ± 0.05] μmol/L; PFC: [0.94 ± 0.08 vs. 0.26 ± 0.08] μmol/L; SAC: [0.57 ± 0.06 vs. 0.15 ± 0.06] μmol/L; SMG: [0.70 ± 0.06 vs. 0.17 ± 0.06] μmol/L). Significant group × task interactions were observed in PMC, S1, FEF, and SMG (Figure 5). Simple effects tests indicated substantial group differences in PMC (*p* = 0.038), S1 (*p* = 0.026), and SMG (p = 0.026) during SLS, with higher activation in the CONT group (PMC: [2.93 ± 0.31 vs. 2.11 ± 0.25] μmol/L; S1: [1.55 ± 0.18 vs. 1.04 ± 0.14] μmol/L; SMG: [0.84 ± 0.10 vs. 0.56 ± 0.08] μmol/L). In the FEF area, the only significant difference was found during the K-FE task (*p* = 0.043), where the ACLR group showed greater activation (0.26 ± 0.06 vs. 0.06 ± 0.07 μmol/L). However, during SLS, no group difference was observed in the FEF area (*p* = 0.152).

**Table 4:** Results of two-way mixed ANOVA for ΔHbO across brain regions during contralateral limb tasks.

	Main group effect		Main task effect		Group × Task interaction effect	
	*F*	*p*	*F*	*p*	*F*	*p*
PMC	0.203	0.653	39.108	<0.001*	6.141	0.013*
S1	0.483	0.487	27.806	<0.001*	5.974	0.015*
FEF	0.178	0.673	13.885	<0.001*	5.973	0.015*
PFC	1.671	0.196	40.518	<0.001*	2.026	0.155
SAC	0.037	0.847	25.131	<0.001*	3.281	0.07
SMG	0.592	0.442	34.406	<0.001*	5.638	0.018*

*indicates significant difference.

### 3.3 Correlation between clinical functions and ΔHbO in brain regions during different tasks in the ACLR group

As shown in Table 5, several significant correlations were observed in the ACLR group. During the K-FE task with the affected limb, increased ΔHbO in PMC, S1, FEF, and PFC was significantly correlated with lower Q-LSI scores (PMC: *r* = −0.38, *p* = 0.032; S1: *r* = −0.363, *p* = 0.041; FEF: *r* = −0.363, *p* = 0.041; PFC: *r* = −0.412, *p* = 0.019). During the K-FE task with the contralateral limb, increased ΔHbO in PMC, S1, and SAC was significantly correlated with a lower IKDC score (PMC: *r* = −0.384, *p* = 0.025; S1: *r* = −0.408, *p* = 0.017; SAC: *r* = −0.367, *p* = 0.033). During the SLS task with the affected limb, increased ΔHbO in FEF was significantly correlated with both lower SLHD-LSI and ACL-RSI (SLH-LSI: *r* = −0.335, *p* = 0.049; ACL-RSI: *r* = −0.455, *p* = 0.006). No significant correlations were found during the SLS task with the contralateral limb. Overall, these findings suggested task-specific and region-specific patterns of altered cortical activation in patients with ACLR, some of which were linked to functional deficits and reduced clinical performance.

**Table 5:** The correlation between clinical functions and ΔHbO in brain areas during different motor tasks [r(p)].

	Brain area	Q-LSI	SLHD-LSI	ACL-RSI	IKDC
Affected limb K-FE	PMC	−0.380*(0.032)	−0.244(0.164)	−0.132(0.457)	−0.337(0.051)
	S1	−0.363*(0.041)	−0.265(0.13)	−0.203(0.249)	−0.331(0.056)
	FEF	−0.349*(0.05)	−0.218(0.217)	−0.1(0.575)	−0.215(0.223)
	PFC	−0.412*(0.019)	−0.302(0.083)	−0.202(0.251)	−0.235(0.182)
	SAC	−0.314(0.08)	−0.203(0.25)	−0.019(0.915)	−0.316(0.068)
	SMG	−0.317(0.077)	−0.145(0.413)	−0.087(0.623)	−0.255(0.146)
Contralateral limb K-FE	PMC	−0.184(0.314)	−0.154(0.386)	−0.046(0.797)	−0.384*(0.025)
	S1	−0.201(0.269)	−0.242(0.169)	−0.017(0.926)	−0.408*(0.017)
	FEF	−0.083(0.654)	−0.188(0.287)	0.036(0.84)	−0.245(0.163)
	PFC	−0.32(0.074)	−0.212(0.228)	0.014(0.938)	−0.318(0.067)
	SAC	−0.259(0.152)	−0.207(0.239)	−0.071(0.688)	−0.367*(0.033)
	SMG	−0.161(0.377)	−0.211(0.232)	−0.141(0.426)	−0.322(0.063)
Affected limb SLS	PMC	−0.103(0.569)	−0.149(0.393)	−0.116(0.508)	0.112(0.521)
	S1	0.066(0.714)	−0.054(0.756)	−0.062(0.724)	0.09(0.608)
	FEF	−0.216(0.226)	−0.335*(0.049)	−0.455**(0.006)	−0.303(0.077)
	PFC	−0.223(0.212)	−0.244(0.158)	−0.112(0.522)	−0.166(0.342)
	SAC	−0.061(0.735)	−0.081(0.644)	−0.256(0.137)	−0.178(0.305)
	SMG	0.027(0.883)	−0.053(0.763)	−0.216(0.212)	−0.087(0.619)
Contralateral limb SLS	PMC	0.031(0.864)	0.086(0.624)	0.024(0.891)	0.099(0.572)
	S1	0.148(0.41)	0.125(0.475)	0.029(0.869)	0.08(0.65)
	FEF	−0.012(0.948)	0.05(0.778)	−0.092(0.597)	−0.16(0.358)
	PFC	0.014(0.937)	−0.046(0.794)	0.179(0.303)	0.016(0.927)
	SAC	−0.002(0.992)	0.068(0.697)	0.079(0.651)	−0.125(0.473)
	SMG	0.174(0.332)	0.072(0.681)	0.067(0.702)	0.003(0.985)

Q-LSI: quadriceps limb symmetry index; SLHD-LSI: Single-Leg hop distance limb distance symmetry index; ACL-RSI: ACL Return to Sport Index; IKDC: International Knee Documentation Committee; PMC, Pre-Motor and Supplementary Motor Cortex; S1, Primary Somatosensory Cortex; FEF, Frontal Eye Fields; PFC, Prefrontal Cortex; SAC, Somatosensory Association Cortex; SMG, Supramarginal Gyrus; * indicates statistical significance.

## 4 Discussion

The primary finding of this study was that brain activation patterns differed based on both group and task. The ACLR group exhibited brain activation patterns distinct from healthy controls during the transition from a simple (K-FE) to a complex (SLS) task. Furthermore, the correlation results provided insight into the role of altered brain activation patterns following ACLR during complex motor tasks, supporting the presence of newly adopted neural and contralateral compensatory strategies.

Regarding the main effect of group, S1 and SMG activation in the ACLR group was lower than that in the CONT group during affected limb tasks, whereas no significant main group difference was observed during contralateral limb tasks. S1 is primarily responsible for processing cutaneous stimulation, especially tactile sensation (Delhaye et al., 2018), and the SMG is involved in spatial sensory processing and integrating sensory processing (Ben-Shabat et al., 2015). Damage to proprioceptors likely reduces afferent sensory information, leading to lower activation in these regions during tasks with the affected limb. However, previous studies have found increased somatosensory cortex activation in patients with ACLR (Baumeister et al., 2008; Schnittjer et al., 2023). This may be due to the blockage of visual afferent information in prior studies due to fMRI limitations or proprioceptive task demands. Without visual input, proprioceptive deficits increase the demands of neural processing, resulting in higher sensory cortex activation. Sherman et al. (2023) showed that somatosensory activation in patients with ACLR was lower in normal visual conditions. Thus, visual input significantly influences somatosensory activation patterns post-ACLR, likely due to inhibition and reweighting of neural resources to compensate for motor deficits (Grooms et al., 2017; Lehmann et al., 2021; Sherman et al., 2023).

Regarding the main effect of task difficulty, participants exhibited greater brain activation during the SLS task than during the K-FE task. The K-FE is widely used to assess brain plasticity post-ACLR (Baumeister et al., 2008; Grooms et al., 2017; Lepley et al., 2019). SLS involves the same knee joint movement as K-FE but requires the simultaneous maintenance of balance and speed. The proposed method is similar to the single-leg hop, which is a commonly used functional test; however, it offers a safer alternative (Ageberg and Cronström, 2018). The validity and reliability of SLS for assessing motor function after ACLR have been well established (Cardoso et al., 2021). Therefore, the increased cortical activity during SLS revealed that more neural activation was required to perform a more challenging task.

During the task with the affected limb, compared with the CONT group, the ACLR group showed reduced SMG activation during SLS. As the SMG is involved in coordinating limb-environment interactions (Ben-Shabat et al., 2015), reduced activation might be induced by ligament damage or compensatory strategies. Compared with simple tasks, complex tasks like SLS require more afferent sensory input to sustain balance, which is limited when ligament deficits induce degraded proprioception. While some prior research noted increased motor and sensory cortex activation (An et al., 2022) or visual compensatory mechanisms (Grooms et al., 2017; Criss et al., 2020), these may reflect compensatory mechanisms aimed at maintaining motor output. Such strategies are cognitively demanding and can limit the brain’s capacity (Constantinidis and Klingberg, 2016), which led to the non-significant increment of brain activation in ACLR during the SLS task. However, during the K-FE task with the affected limb, other studies found that the motor, sensory, and cognitive brain areas showed significantly different activation in patients with ACLR compared with healthy controls or their contralateral legs (Baumeister et al., 2008; Grooms et al., 2017; Lepley et al., 2019; Schnittjer et al., 2023). Our study’s results were not consistent with previous studies, which may be explained by the relatively low difficulty of the task, the proximity of participants to the recommended return-to-sport timeline, and their engagement in a structured online rehabilitation program.

During the task with the contralateral limb, PMC, S1, and SMG showed similar interaction effects as observed during the task with the affected limb, indicating that the altered cortical activation pattern following ACLR also affected the contralateral limb tasks. Because the knee of the contralateral limb was intact, the lack of activation in ACLR during SLS was mainly induced by limited neural resources for complex tasks, followed by new cortical activation adaptation. However, it is interesting to find that patients with ACLR had greater brain area activation than the CONT group during the K-FE task with the contralateral limb. The results indicated that more neural resources were needed for patients with ACLR during easy tasks, even for the intact limb. The activity of FEF neurons is associated with essential eye movements for visual tasks (Vernet et al., 2014), and the significantly greater activation of FEF neurons suggests the adoption of a visual compensation strategy in patients with ACLR. As suggested by Paterno et al. (2011), the contralateral limb played a more active role in simple tasks post-ACLR. However, as task difficulty increases, neural resources may become insufficient to support additional compensatory control, thereby limiting the compensatory strategy of the contralateral limb.

Finally, the correlation between cortical activity and clinical function supported our hypothesis. During the simple task with the affected limb, Q-LSI was negatively correlated with activation in PMC, S1, FEF, and PFC. Criss et al. (2023) also noted this, although they reported higher brain activation in patients with ACLR, suggesting that as quadriceps strength improves, brain activation becomes more efficient. Although the cortical activity of our study did not show a significant difference between the two groups during a simple task with the affected limb, the negative relationship in our study also indicated that the rehabilitation process may contribute to more efficient brain activation because K-FE is the most common training exercise for restoring lower extremity strength. During the SLS task involving the affected limb, the SLHD-LSI and ACL-RSI were negatively correlated with FEF activation, indicating that better physical and psychological function reduced reliance on visual compensation. These findings align with the above findings that compensatory strategies could consume neural resources and impair performance (Buschman et al., 2011). IKDC scores were inversely related to PMC, S1, and SAC activation during contralateral K-FE tasks. This suggests that improved subjective outcomes correspond with reduced compensatory brain activation, reinforcing the idea that reduction of contralateral compensatory engagement will increase the knee’s subjective function.

## 5 Limitation

This study has several limitations that should be considered. First, due to its cross-sectional design, the observed differences in brain activation may not be entirely attributable to ACLR, as some neural patterns could have existed prior to injury or surgery. Future research should consider a longitudinal design to better capture neural changes throughout recovery following ACL injury and reconstruction. Second, the sample size of the two groups was unequal, and some confounding variables were not controlled due to real-world recruitment constraints. Although this may affect the reliability of the findings, statistical guidelines indicate that ANOVA and GLM analyses are generally robust to unequal group sizes (Field, 2013; Dobson and Barnett, 2018). Furthermore, there was no statistically significant difference in baseline demographic characteristics between the groups, supporting the comparability of the cohorts. Nevertheless, future studies should aim to control for additional factors, particularly limb dominance and injury side, to minimize the potential effects of brain lateralization. Finally, fNIRS measures cortical activity and covers only a limited portion of the brain. However, its portability allows real-time brain activation measurements during functional motor tasks in patients with ACLR. Future studies should consider expanding the optode layout to include the visual cortex to provide further insight into compensatory strategies.

### 5.1 Clinical significance

Patients with ACLR appear to develop new neural compensatory strategies during complex tasks, which may result in limited cerebral resources and hinder motor function recovery. Understanding these compensatory mechanisms is crucial for optimizing rehabilitation programs. The role of the contralateral limb in compensation also requires further investigation. This study supports the validity and reliability of fNIRS for detecting neuroplastic changes during real motor tasks in patients with ACLR, consistent with previous research. However, simple tasks may not be adequate for capturing meaningful brain activation using fNIRS in this population.

## 6 Conclusion

This study highlighted the necessity of using different motor tasks to examine brain activation patterns in patients with ACLR. The findings demonstrated reduced sensory cortex activation during movement in this population. Moreover, more complex tasks require greater neural activation overall. In response to increased task complexity, patients with ACLR exhibited significantly lower brain activation compared with healthy controls, likely due to cerebral resource limitations associated with compensatory strategies. Although the contralateral limb appeared to assist with task performance, this compensatory effect diminished as task complexity increased.

## Data Availability

The original contributions presented in the study are included in the article/Supplementary material, further inquiries can be directed to the corresponding author.
