# Supplementary material for: Requirement of a complex motor task to identify neuroplastic changes in motor control of the lower extremity in patients with anterior cruciate ligament reconstruction: a fNIRS study
Source: Front Hum Neurosci. 2025 Jul 10;19:1595284. doi: 10.3389/fnhum.2025.1595284 (PMC12288510; doi:10.3389/fnhum.2025.1595284)
Supplement: Supplementary file 1 [file Supplementary_file_1.docx]

Supplementary 1

1. Montreal Neurological Institute (MNI) coordinates for 20 channels and 16 optodes

| S1 | -39.835 | -9.543 | 89.911 |
| --- | --- | --- | --- |
| S2 | -63.1 | 23.879 | 52.682 |
| S3 | -69.286 | -48.011 | 65.668 |
| S4 | -84.098 | -13.846 | 24.756 |
| S5 | 38.717 | -8.599 | 90.282 |
| S6 | 62.315 | 24.986 | 53.08 |
| S7 | 69.044 | -46.885 | 66.427 |
| S8 | 83.687 | -11.831 | 25.487 |
| D1 | -35.971 | 27.644 | 77.821 |
| D2 | -39.429 | -48.345 | 92.764 |
| D3 | -70.634 | -11.463 | 63.077 |
| D4 | -77.435 | 19.001 | 18.879 |
| D5 | 35.174 | 28.261 | 77.967 |
| D6 | 38.78 | -47.709 | 93.218 |
| D7 | 69.701 | -9.782 | 63.617 |
| D8 | 76.997 | 20.379 | 19.465 |
| CH01 | -38.473 | 8.634 | 84.604 |
| CH02 | -39.895 | -28.88 | 93.294 |
| CH03 | -57.076 | -9.562 | 77.439 |
| CH04 | -51.155 | 26.914 | 66.065 |
| CH05 | -67.497 | 6.477 | 58.072 |
| CH06 | -71.835 | 22.115 | 37.64 |
| CH07 | -56.069 | -49.118 | 80.686 |
| CH08 | -72.341 | -29.617 | 65.522 |
| CH09 | -80.047 | -11.793 | 44.03 |
| CH10 | -81.342 | 2.605 | 21.617 |
| CH11 | 37.492 | 9.942 | 84.648 |
| CH12 | 39.401 | -28.293 | 93.506 |
| CH13 | 55.859 | -8.634 | 78.224 |
| CH14 | 49.824 | 27.354 | 67.243 |
| CH15 | 66.337 | 8.158 | 58.736 |
| CH16 | 71.344 | 23.106 | 37.681 |
| CH17 | 54.798 | -47.767 | 82.138 |
| CH18 | 71.228 | -27.778 | 67.002 |
| CH19 | 79.353 | -10.899 | 45.643 |
| CH20 | 80.866 | 4.38 | 23.264 |

1. Corresponding Broadman area and percentage of overlap for 20 channels and 16 optodes

CH01 : 6 - Pre-Motor and Supplementary Motor Cortex, 1

CH02 : 1 - Primary Somatosensory Cortex, 0.084967

CH02 : 2 - Primary Somatosensory Cortex, 0.18627

CH02 : 3 - Primary Somatosensory Cortex, 0.3268

CH02 : 4 - Primary Motor Cortex, 0.34641

CH02 : 5 - Somatosensory Association Cortex, 0.026144

CH02 : 6 - Pre-Motor and Supplementary Motor Cortex, 0.029412

CH03 : 1 - Primary Somatosensory Cortex, 0.023077

CH03 : 3 - Primary Somatosensory Cortex, 0.32308

CH03 : 4 - Primary Motor Cortex, 0.14615

CH03 : 6 - Pre-Motor and Supplementary Motor Cortex, 0.50769

CH04 : 6 - Pre-Motor and Supplementary Motor Cortex, 0.42339

CH04 : 8 - Includes Frontal eye fields, 0.57661

CH05 : 3 - Primary Somatosensory Cortex, 0.0040161

CH05 : 4 - Primary Motor Cortex, 0.072289

CH05 : 6 - Pre-Motor and Supplementary Motor Cortex, 0.91165

CH05 : 8 - Includes Frontal eye fields, 0.012048

CH06 : 6 - Pre-Motor and Supplementary Motor Cortex, 0.025362

CH06 : 8 - Includes Frontal eye fields, 0.072464

CH06 : 9 - Dorsolateral prefrontal cortex, 0.90217

CH07 : 2 - Primary Somatosensory Cortex, 0.06

CH07 : 5 - Somatosensory Association Cortex, 0.392

CH07 : 7 - Somatosensory Association Cortex, 0.22

CH07 : 40 - Supramarginal gyrus part of Wernicke's area, 0.328

CH08 : 1 - Primary Somatosensory Cortex, 0.031128

CH08 : 2 - Primary Somatosensory Cortex, 0.29961

CH08 : 40 - Supramarginal gyrus part of Wernicke's area, 0.66926

CH09 : 1 - Primary Somatosensory Cortex, 0.16207

CH09 : 2 - Primary Somatosensory Cortex, 0.15862

CH09 : 3 - Primary Somatosensory Cortex, 0.1931

CH09 : 4 - Primary Motor Cortex, 0.093103

CH09 : 6 - Pre-Motor and Supplementary Motor Cortex, 0.38621

CH09 : 40 - Supramarginal gyrus part of Wernicke's area, 0.0068966

CH10 : 1 - Primary Somatosensory Cortex, 0.0063291

CH10 : 3 - Primary Somatosensory Cortex, 0.041139

CH10 : 4 - Primary Motor Cortex, 0.11709

CH10 : 6 - Pre-Motor and Supplementary Motor Cortex, 0.52532

CH10 : 22 - Superior Temporal Gyrus, 0.028481

CH10 : 43 - Subcentral area, 0.28165

CH11 : 6 - Pre-Motor and Supplementary Motor Cortex, 1

CH12 : 1 - Primary Somatosensory Cortex, 0.062092

CH12 : 2 - Primary Somatosensory Cortex, 0.16667

CH12 : 3 - Primary Somatosensory Cortex, 0.34314

CH12 : 4 - Primary Motor Cortex, 0.37582

CH12 : 5 - Somatosensory Association Cortex, 0.052288

CH13 : 3 - Primary Somatosensory Cortex, 0.25095

CH13 : 4 - Primary Motor Cortex, 0.17871

CH13 : 6 - Pre-Motor and Supplementary Motor Cortex, 0.57034

CH14 : 6 - Pre-Motor and Supplementary Motor Cortex, 0.45749

CH14 : 8 - Includes Frontal eye fields, 0.54251

CH15 : 3 - Primary Somatosensory Cortex, 0.033333

CH15 : 4 - Primary Motor Cortex, 0.095833

CH15 : 6 - Pre-Motor and Supplementary Motor Cortex, 0.87083

CH16 : 6 - Pre-Motor and Supplementary Motor Cortex, 0.14286

CH16 : 8 - Includes Frontal eye fields, 0.10902

CH16 : 9 - Dorsolateral prefrontal cortex, 0.74812

CH17 : 2 - Primary Somatosensory Cortex, 0.040486

CH17 : 5 - Somatosensory Association Cortex, 0.45344

CH17 : 7 - Somatosensory Association Cortex, 0.35223

CH17 : 40 - Supramarginal gyrus part of Wernicke's area, 0.15385

CH18 : 1 - Primary Somatosensory Cortex, 0.067925

CH18 : 2 - Primary Somatosensory Cortex, 0.24528

CH18 : 40 - Supramarginal gyrus part of Wernicke's area, 0.68679

CH19 : 1 - Primary Somatosensory Cortex, 0.26027

CH19 : 2 - Primary Somatosensory Cortex, 0.16781

CH19 : 3 - Primary Somatosensory Cortex, 0.16096

CH19 : 4 - Primary Motor Cortex, 0.11644

CH19 : 6 - Pre-Motor and Supplementary Motor Cortex, 0.26712

CH19 : 40 - Supramarginal gyrus part of Wernicke's area, 0.027397

CH20 : 3 - Primary Somatosensory Cortex, 0.092063

CH20 : 4 - Primary Motor Cortex, 0.08254

CH20 : 6 - Pre-Motor and Supplementary Motor Cortex, 0.6

CH20 : 43 - Subcentral area, 0.2254

S1 : 4 - Primary Motor Cortex, 0.1

S1 : 6 - Pre-Motor and Supplementary Motor Cortex, 0.9

S2 : 6 - Pre-Motor and Supplementary Motor Cortex, 0.22568

S2 : 8 - Includes Frontal eye fields, 0.66537

S2 : 9 - Dorsolateral prefrontal cortex, 0.10895

S3 : 40 - Supramarginal gyrus part of Wernicke's area, 1

S4 : 1 - Primary Somatosensory Cortex, 0.16358

S4 : 2 - Primary Somatosensory Cortex, 0.21605

S4 : 3 - Primary Somatosensory Cortex, 0.083333

S4 : 4 - Primary Motor Cortex, 0.0092593

S4 : 6 - Pre-Motor and Supplementary Motor Cortex, 0.027778

S4 : 40 - Supramarginal gyrus part of Wernicke's area, 0.28086

S4 : 42 - Primary and Auditory Association Cortex, 0.0092593

S4 : 43 - Subcentral area, 0.20988

S5 : 4 - Primary Motor Cortex, 0.093333

S5 : 6 - Pre-Motor and Supplementary Motor Cortex, 0.90667

S6 : 6 - Pre-Motor and Supplementary Motor Cortex, 0.28063

S6 : 8 - Includes Frontal eye fields, 0.67589

S6 : 9 - Dorsolateral prefrontal cortex, 0.043478

S7 : 40 - Supramarginal gyrus part of Wernicke's area, 1

S8 : 1 - Primary Somatosensory Cortex, 0.16

S8 : 2 - Primary Somatosensory Cortex, 0.20923

S8 : 3 - Primary Somatosensory Cortex, 0.11077

S8 : 6 - Pre-Motor and Supplementary Motor Cortex, 0.027692

S8 : 40 - Supramarginal gyrus part of Wernicke's area, 0.37231

S8 : 43 - Subcentral area, 0.12

D1 : 6 - Pre-Motor and Supplementary Motor Cortex, 0.94118

D1 : 8 - Includes Frontal eye fields, 0.058824

D2 : 2 - Primary Somatosensory Cortex, 0.020619

D2 : 5 - Somatosensory Association Cortex, 0.51546

D2 : 7 - Somatosensory Association Cortex, 0.46392

D3 : 1 - Primary Somatosensory Cortex, 0.21429

D3 : 2 - Primary Somatosensory Cortex, 0.22222

D3 : 3 - Primary Somatosensory Cortex, 0.39286

D3 : 4 - Primary Motor Cortex, 0.10317

D3 : 6 - Pre-Motor and Supplementary Motor Cortex, 0.06746

D4 : 6 - Pre-Motor and Supplementary Motor Cortex, 0.19094

D4 : 9 - Dorsolateral prefrontal cortex, 0.20388

D4 : 44 - pars opercularis, part of Broca's area, 0.42395

D4 : 45 - pars triangularis Broca's area, 0.18123

D5 : 6 - Pre-Motor and Supplementary Motor Cortex, 0.93818

D5 : 8 - Includes Frontal eye fields, 0.061818

D6 : 2 - Primary Somatosensory Cortex, 0.017007

D6 : 5 - Somatosensory Association Cortex, 0.4966

D6 : 7 - Somatosensory Association Cortex, 0.48639

D7 : 1 - Primary Somatosensory Cortex, 0.2753

D7 : 2 - Primary Somatosensory Cortex, 0.14575

D7 : 3 - Primary Somatosensory Cortex, 0.48178

D7 : 4 - Primary Motor Cortex, 0.08502

D7 : 6 - Pre-Motor and Supplementary Motor Cortex, 0.0080972

D7 : 40 - Supramarginal gyrus part of Wernicke's area, 0.0040486

D8 : 6 - Pre-Motor and Supplementary Motor Cortex, 0.2349

D8 : 9 - Dorsolateral prefrontal cortex, 0.27517

D8 : 44 - pars opercularis, part of Broca's area, 0.3255

D8 : 45 - pars triangularis Broca's area, 0.16443
